# Laryngeal edema is common in HAE and demands preventive measures

**DOI:** 10.1186/1939-4551-8-S1-A190

**Published:** 2015-04-08

**Authors:** Cristine Secco Rosario, Ana Flavia Machado, Jose Eduardo Thomaz, Camila Piragine, Alessandra Bitencourt, Isabela D'agulham, Herberto Jose Chong Neto, Carlos Antônio Riedi, Nelson Rosario Filho

**Affiliations:** 1 Federal University of Paraná, Brazil

## Background

The aim of this study was to show the clinical characteristics and treatment of Hereditary Angioedema (HAE) in a tertiary center from Curitiba, south of Brazil.

## Methods

A cross-sectional study reviewing records of patients in the Hospital de Clínicas, Federal University of Paraná. We analised the clinical characteristics, laboratory tests and treatment of patients with HAE.

## Results

Forty two patients, male (45%), age mean 27.1±16.9 years. Symptoms started at median age 14 years (range 1 to 58ys.). Thirty seven (86%) had familial history of HAE. They had a median of 2 episodes/mo (range 0.2 to 30) lasting 3±1.7 days/episode. Edema of limbs, face, genital and laryngeal, abdominal pain, diarrhea and vomiting were seen in 73.8%, 52.4%, 23.8%, 17%, 81%, 24% and 38.1%, respectively. Twenty four (57%) had low C4 level, mean serum level of C4=12.7±10.6 mg/dL; 26 (62%) had low levels of C1 esterase inhibitor, 4 (9.5%) had low functional C1 esterase inhibitor and 12 (28.6%) had C1q deficiency and 3 (7.1%) had HAE type III confirmed. Fifteen (28.9%) were treated with Danazol 175±134 mg/day, 15 (28.9%) were using tranexamic acid as needed. No patient had Icatibant access.

## Conclusions

HAE is a difficult to control disease. Patients needs to have a facilitated access to newer therapies to control their symptoms and reduce the adverse events of Danazol.

